# Preparation of Protein Aerogel Particles for the Development of Innovative Drug Delivery Systems

**DOI:** 10.3390/gels8120765

**Published:** 2022-11-24

**Authors:** Daria Lovskaya, Anna Bezchasnyuk, Maria Mochalova, Pavel Tsygankov, Artem Lebedev, Yana Zorkina, Eugene Zubkov, Aleksandra Ochneva, Olga Gurina, Artemiy Silantyev, Alexander Majouga, Natalia Menshutina

**Affiliations:** 1 Department of Chemical and Pharmaceutical Engineering, Mendeleev University of Chemical Technology, 125047 Moscow, Russia; 2 Department of Basic and Applied Neurobiology, V. Serbsky Federal Medical Research Centre of Psychiatry and Narcology, Kropotkinsky Per. 23, 119034 Moscow, Russia

**Keywords:** aerogel, protein, nasal drug delivery system, clomipramine, in vivo evaluations, pharmacokinetics, bioavailability

## Abstract

The research was oriented towards the preparation of aerogel particles based on egg white and whey protein isolate using various dispersion methods: dripping, spraying, and homogenization. Based on the results of analytical studies, the most appropriate samples were selected to obtain aerogels loaded with the drug. The results of the experimental research were used to study methods for obtaining nasal drug delivery systems based on aerogels. Protein aerogels were obtained by thermal gelation followed by supercritical drying. The obtained particles of protein aerogels have a specific surface area of up to 350 m^2^/g with a pore volume of up to 2.9 cm^3^/g, as well as a porosity of up to 95%. The results of experimental studies have shown that changing the dispersion method makes it possible to control the structural characteristics of protein aerogel particles. The results of the studies were applied to obtain innovative nasal drug delivery systems for the treatment of socially significant diseases. Analytical studies were conducted to determine the amount and state of adsorbed drugs in protein aerogel particles, as well as in vivo experiments on the distribution of clomipramine in blood plasma and brain tissue of rats to study the pharmacokinetics and bioavailability of the resulting drug-loaded protein aerogel.

## 1. Introduction

The possibility of using various biopolymer materials to obtain aerogels and their further potential application in the pharmaceutical, food, and biomedical industries are of high interest to modern science [1,2,3]. Recently, biopolymer aerogels have become particularly relevant in the production of new drug delivery systems, for example, to obtain pharmaceutical compositions with improved pharmacokinetic properties [4]. The creation of drug delivery systems using protein aerogels makes it possible to obtain an effective material with specified properties [5]. Protein aerogels have a number of characteristics of organic aerogels, such as high values of specific surface area (up to 478 m^2^/g) and porosity (up to 99.9%), along with low density (0.003–0.5 g/cm^3^), and due to unique properties of the protein, it is possible to obtain a biocompatible and biodegradable material [6].

Such properties of protein aerogel particles as size, shape, density, pore size, specific surface area, and its functionality can affect the characteristics of the pharmaceutical composition, for example, the adsorption process and, consequently, the maximum amount of the drug loaded into the aerogel. The scope of application of such material for a particular case determines the size of the aerogel particles. Thus, for the development of drug-loaded compositions for nasal applications based on peptides, for example, a range of particle sizes of 10–45 microns may be optimal [7]. For oral delivery, for example, carriers of vitamin supplements, particles in a wide range of sizes from nanometers to several centimeters may be required [8,9,10,11]. However, there are no studies of fine-tuning the structural characteristics of protein aerogels. To solve this problem, it is necessary to study the processes of obtaining protein aerogel particles by various dispersion methods: dripping, spraying, and homogenization. There are a large number of works in the literature devoted to the choice of an effective method for obtaining protein gel particles [12,13,14]. It was demonstrated in [15,16] that the formation of gels is possible during the thermal denaturation of proteins, from which aerogels can be obtained using a supercritical drying process.

One of the methods for obtaining protein aerogel particles is thermal gelation followed by drying of the gels in a supercritical carbon dioxide medium. Carbon dioxide has relatively low critical points (its critical temperature is 31.1 °C, and the pressure is 73.8 atm) and a low price; its use also complies with all of the principles of “green” chemistry [17]. Supercritical drying of gels allows for the preservation of their original structure and obtaining of highly porous materials with a three-dimensional internal structure [18,19,20]. The control of various parameters affecting the structural and physicochemical properties of protein aerogel particles has been the subject of ever-increasing interest over the past ten years. The use of various methods of dispersing the protein solution into oil during gelation can become an effective method of directional regulation of the internal structure and particle size distribution.

The use of aerogels as delivery systems for a drug is a particularly promising direction, because there is a large class of compounds that need to be loaded into the aerogel for controlled release and improved bioavailability, increased effectiveness, and minimization of side effects [21,22,23].

The drug can be loaded into the aerogel using various methods [5,24,25], for example, at the stage of obtaining a biopolymer solution [25], at the solvent exchange stage [25], the method of adsorption from a solution [24,25] and the method of supercritical adsorption [5,24].

The method of loading the drug at the preparation stage is to add the drug to the biopolymer solution before the gelation stage. In this way, it is possible to obtain materials where the drug is chemically or physically bound to the solid phase [23,26,27]. There are a number of limitations for carrying out this loading method. The medicine should dissolve well in the liquid phase of the biopolymer solution. A significant advantage in this case will be the interaction of the drug with the biopolymer forming the hydrogel skeleton. The next important limitation is the insolubility of the drug in the solvent used to prepare the gel for supercritical drying. In addition, the drug must remain stable during the supercritical drying process.

The drug is loaded at the solvent replacement stage by filling the pores of the hydrogel with a solution of the drug in a solvent used to prepare the gel for supercritical drying [28,29]. This method consists of using a supercritical fluid as an anti-solvent. To successfully load the drug into the aerogel using this method, it is necessary that the drug be dissolved in the liquid used to prepare the gel for supercritical drying, and be insoluble (slightly soluble) in the supercritical fluid. Another limitation for this method is the drug must remain stable during the supercritical drying process.

The supercritical adsorption process has a number of advantages. Due to the use of carbon dioxide as a supercritical liquid, the process proceeds using a non-toxic solvent, which is important when obtaining drug delivery systems; the principles of “green” chemistry are observed. In addition, the parameters of supercritical adsorption (temperature, pressure, time) can be selected in accordance with the stability of the drug. Depending on the matrix and the drug, both physical and chemical adsorption is possible, which increases the percentage of mass loading of the drug and does not damage the internal structure of the aerogel [30,31,32]. Limitations for the use of this method are the solubility of the drug in a supercritical fluid, and the thermal stability of the drug at supercritical parameters.

Adsorption from a solution is the easiest way to load a drug into an aerogel. In this method, the aerogel is immersed in a solution of a drug of a certain concentration for a time [7,31,33]. The aerogel begins to absorb the drug from the solution, which allows for the achievement of sufficiently high concentrations. Typically, this process is followed by air-drying, thermal-drying, or freeze-drying. This method has no limitations on temperature or other parameters. Thus, all types of drugs can be loaded into the aerogel using this method. However, upon contact with a liquid, the structure of most hydrophilic aerogels begins to collapse. Analyzing the reference data on the physicochemical properties of the drug, one can choose one or another method of loading the drug into protein aerogel particles.

Due to the high porosity and low density, drug-loaded aerogels can be used in the form of dry powder for nasal and pulmonary use [1,33,34]. For example, a nasal drug delivery system based on aerogels may become a promising method to deliver drugs directly to the brain. The possibility of targeted drug delivery by the “Nose-to-brain” mechanism largely depends on the properties of the drug delivery system and its ability to reach the target areas of the nasal cavity [35]. Nasal drug delivery, in comparison with other routes of administration, offers advantages such as rapid adsorption of the drug, rapid onset of action, and delivery to the brain bypassing the blood–brain barrier (BBB) [36]. Since lower concentrations are required to achieve a therapeutic dosage than with, for example, the oral administration of drugs, this method of delivery is economically advantageous, and reduces the toxicity of substances [37]. It is important to note that, in order to deliver the drug by the “Nose-to-brain” mechanism, it is necessary that the drug settles on the olfactory area of the nasal cavity [36]. To do this, the carrier matrix must have a certain aerodynamic diameter (from 50 to 100 microns) [7]. Due to the possibility of directional control of characteristic and particle size of aerogels, as well as their low density and high porosity, it is possible to achieve this goal.

The main purpose of this work is to obtain aerogel particles based on egg white and whey protein isolate using various methods of dispersion: drip, spraying, and homogenization. Then, select a matrix for the loading of the drug based on analytical studies of the materials obtained. Finally, study a method for obtaining a nasal drug delivery system based on aerogels.

## 2. Results and Discussion

### 2.1. Scanning Electron Microscopy of Protein Aerogel Particles

**Figure 1:**
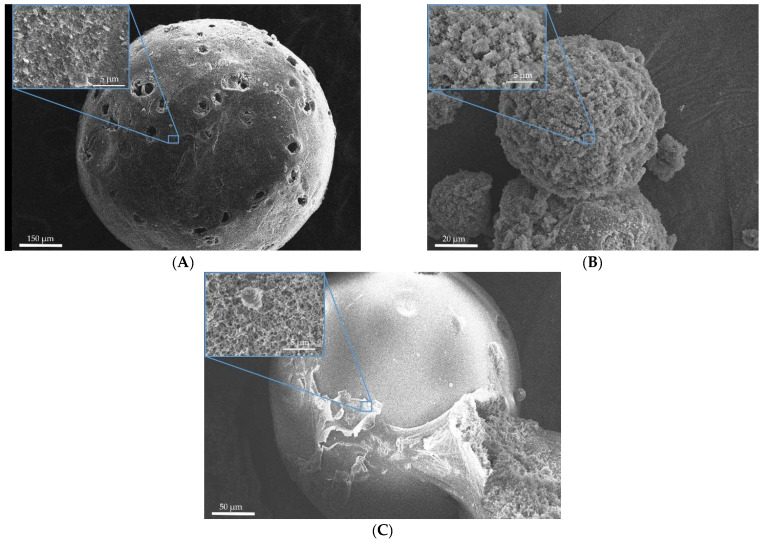
SEM images of the surface of the particles of aerogels based on egg white: (**A**)—sample 1; (**B**)—sample 2; (**C**)—sample 3.

**Figure 2:**
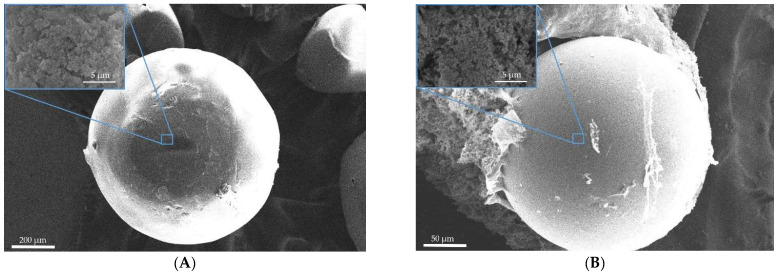

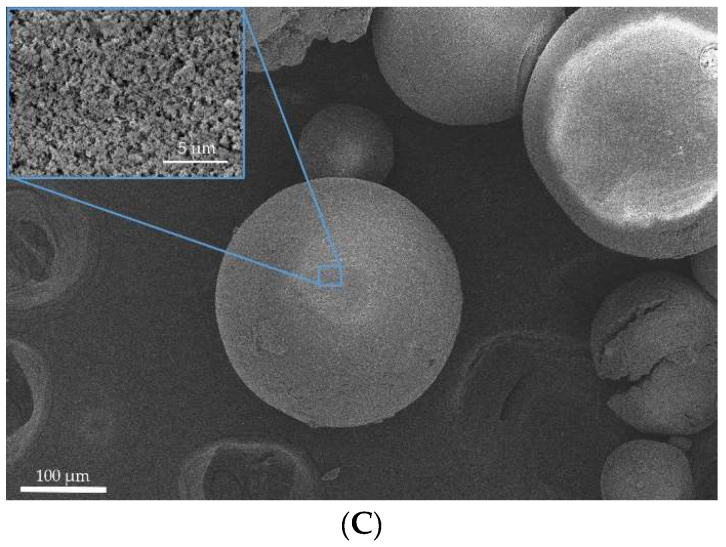
SEM images of the surface of the particles of aerogels based on whey protein isolate: (**A**)—sample 4; (**B**)—sample 5; (**C**)—sample 6.

Figure 1 and Figure 2 show SEM images of the surface of aerogel particles based on egg and whey protein isolate.

The SEM images show that the use of all methods leads to the formation of aerogel particles based on egg white with a surface relief structure with virtually no surface deformation (Figure 1). Samples 4, 5, and 6 (Figure 2), obtained by whey protein isolate, were of spherical shape with the presence of irregularly shaped particles. It can be concluded that, regardless of the method of dispersion, thermal gelation led to the formation of spherical particles of protein aerogel.

### 2.2. Textural Characterization of the Obtained Aerogel Particles

Table 1 presents the following results of analytical studies of protein aerogel particles: specific surface area (S_BET_), average pore volume (V_BJH_), pore diameter (D_pore_), ρ_skeletal_, ρ_bulk_, porosity (P), and average diameters of the particles (d).

The results of analytical studies demonstrate the special properties of protein aerogels. All samples have high porosity with a pore diameter in the range of 2–50 nm (mesoporous material). According to the data presented, it can be seen that the particles have a wide range of sizes and densities, which makes it possible to obtain materials with the tailor-made properties after varying process parameters. Samples based on egg white and whey protein isolates have a high specific surface area (up to 310 and 152 m^2^/g) and a small pore diameter. It is shown that the choice of the dispersion method of the protein solution into oil has a key influence on the size of the final material. The most important is the detection of the relationship between the considered process parameters and the properties of the final materials, and the establishment of certain process parameters to obtain materials with the necessary functional characteristics.

Each of the methods of forming protein gel particles has its advantages and disadvantages, such as cost, simplicity, scaling, volume of production, and the ability to obtain tailor-made protein aerogel particles. Therefore, depending on the application of the material, one or another method of obtaining protein aerogel particles can be used.

It is known that the specific surface area, density, and pore size are the main structural factors affecting the mass loading of a drug [25,26,30]. A higher surface area can lead to a higher mass loading of the drug. In addition, smaller pore sizes can lead to an increase in mass loading due to the existing capillary forces holding drug molecules inside the pores. Highly porous materials can provide efficient mass loading of substances, which is ideal for numerous aerogel applications related to the delivery of drugs.

Further, based on the results obtained, studies were conducted to obtain drug-loaded protein aerogel. Sample 3 was used for this, since its aerodynamic diameter is in the required range (from 50 to 100 microns). Furthermore, sample 3 had one of the highest specific surface areas among the samples with the necessary aerodynamic diameter.

### 2.3. Results of Measurement of the Quantity of Adsorbed Substance by High-Performance Liquid Chromatography

**Figure 3:**
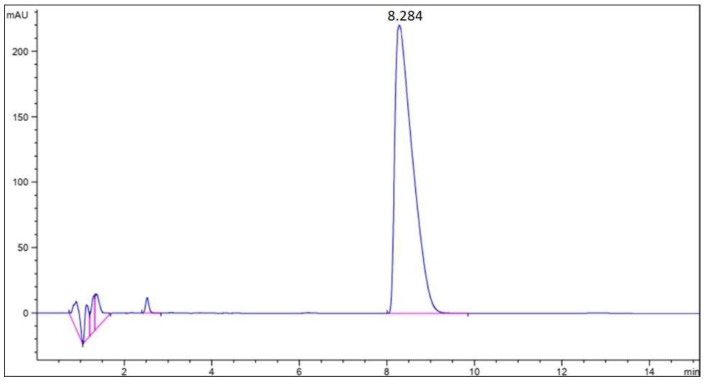
Chromatographic curve of the clomipramine-loaded protein aerogel.

To obtain the concentration of clomipramine in the analyzed solution, the area under the chromatographic curve was recalculated along the calibration line. Figure 3 shows the chromatographic curve of clomipramine.

The results obtained showed that the average mass loading of clomipramine in sample 3 was 14.88 wt.%. According to the data obtained, it is clear that the contact of protein aerogel particles with the drug solution after the supercritical drying stage (adsorption from solution) is an effective way to load the drug. However, this method has its drawbacks for various aerogels, especially for silica aerogels, since they are hydrophilic and break down when immersed in an aqueous medium. In the case of egg protein aerogels, the structure of the aerogel does not collapse when placed in an aqueous medium due to the presence of disulfide bonds in the structure.

### 2.4. Results of X-ray Analysis of the Clomipramine-Loaded Protein Aerogel

**Figure 4:**
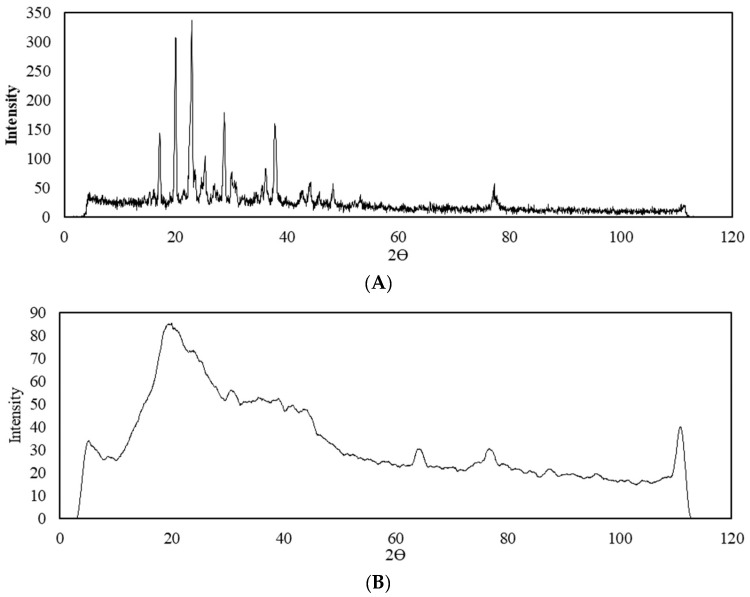
X-ray diffraction patterns: (**A**)—crystalline clomipramine; (**B**)—clomipramine-loaded protein aerogel.

A qualitative X-ray analysis was performed to detect standard crystalline or amorphous phases of clomipramine in the clomipramine-loaded protein aerogel. Figure 4 shows X-ray diffraction patterns of standard clomipramine and the clomipramine-loaded protein aerogel.

Analytical experiments by HPLC proved the presence of clomipramine in the pores of the aerogel in sufficient quantity for analytical study by X-ray analysis. The X-ray diffraction patterns of the clomipramine-loaded protein aerogel does not show peaks corresponding to the crystalline state of clomipramine. Thus, it can be concluded that in the protein aerogel, clomipramine is predominantly in an amorphous state, due to which its release rate can be improved, and thereby a faster therapeutic effect can be achieved.

### 2.5. In Vivo Evaluations

The study included in vivo studies on rats to investigate the accumulation in brain tissue of clomipramine loaded into the protein aerogel particles compared to the pure substance. The effect of chronic administration of clomipramine on the behavior of rats was also evaluated. The experiments were carried out at the V.P. Serbsky National Medical Research Center for Psychiatry and Narcology.

**Figure 5:**
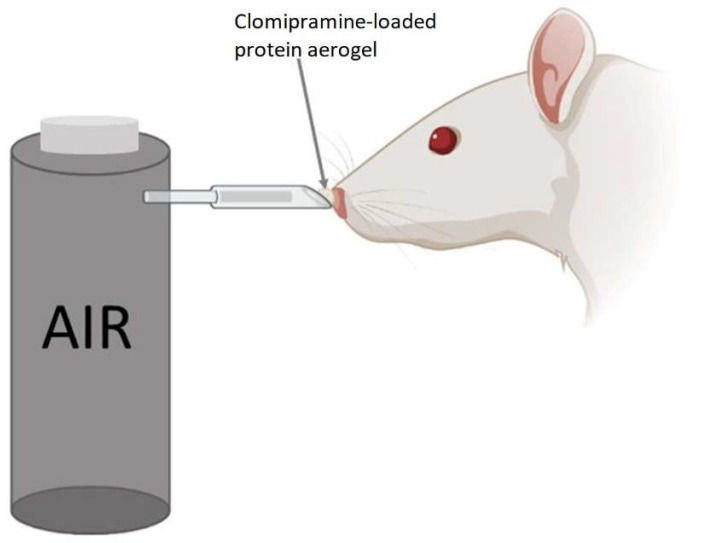
Method of nasal administration of the clomipramine-loaded protein aerogel for rats.

The protein aerogel with clomipramine substance was weighed for each rat before it was administered to the animal. The scheme of administering the drug is shown in Figure 5.

The method of delivering aerogel to the rat’s nose is similar to the methods described in the works [38,39,40], but has several improvements. The powdered substance was placed in a cone-shaped tip, which was connected to a tube from a compressed air container. The cone-shaped tip was placed in the rat’s nostril, and at the same time, we pressed the button to supply air from the container. Half of the dose was administered in one nostril and the other half in the other nostril.

#### 2.5.1. Behavioral Experiment

Statistically significant differences were shown in the sucrose preference test (F = 7.01; *p* = 0.004), the new object recognition test (F = 6.5; *p* = 0.006), and the forced swim test (F = 46; *p* < 0.001).

**Figure 6:**
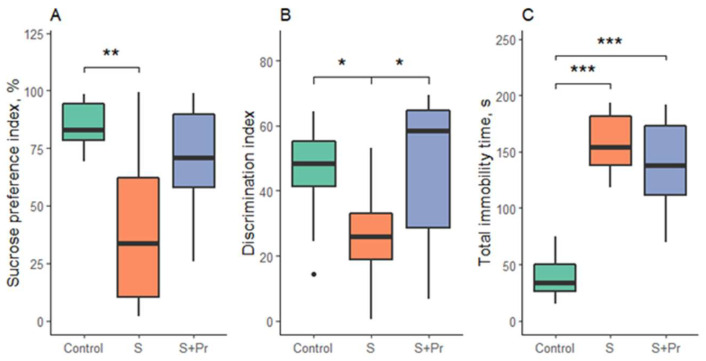
Behavioral test results: (**A**)—sucrose preference test; (**B**)—new object recognition test; (**C**)—forced swim test. S-chronic ultrasound stress group, S + Pr—group of rats exposed to chronic ultrasound stress with protein aerogel nasal treatment. Black circles—outliers; *—*p* < 0.05, **—*p* < 0.01, ***—*p* < 0.001.

Testing revealed the appearance of anhedonia (*p* = 0.003), decreased index of new object recognition (*p* = 0.01), and increased immobility in the forced swimming test (*p* < 0.001) in the group of rats after chronic ultrasound stress without drug treatment, compared with the control. After treatment with the protein aerogel containing clomipramine, there was a partial recovery of anhedonia in some rats, but no statistically significant differences with the stress and control groups were observed. There was a recovery of cognitive functions in the “New Object Recognition” test to the value in the control rats (*p* = 0.02 compared to the stress group without treatment). Immobility in rats treated with the protein aerogel containing clomipramine remained at the level of untreated stressed animals (Figure 6).

#### 2.5.2. Distribution of Clomipramine in Brain Tissue

**Figure 7:**
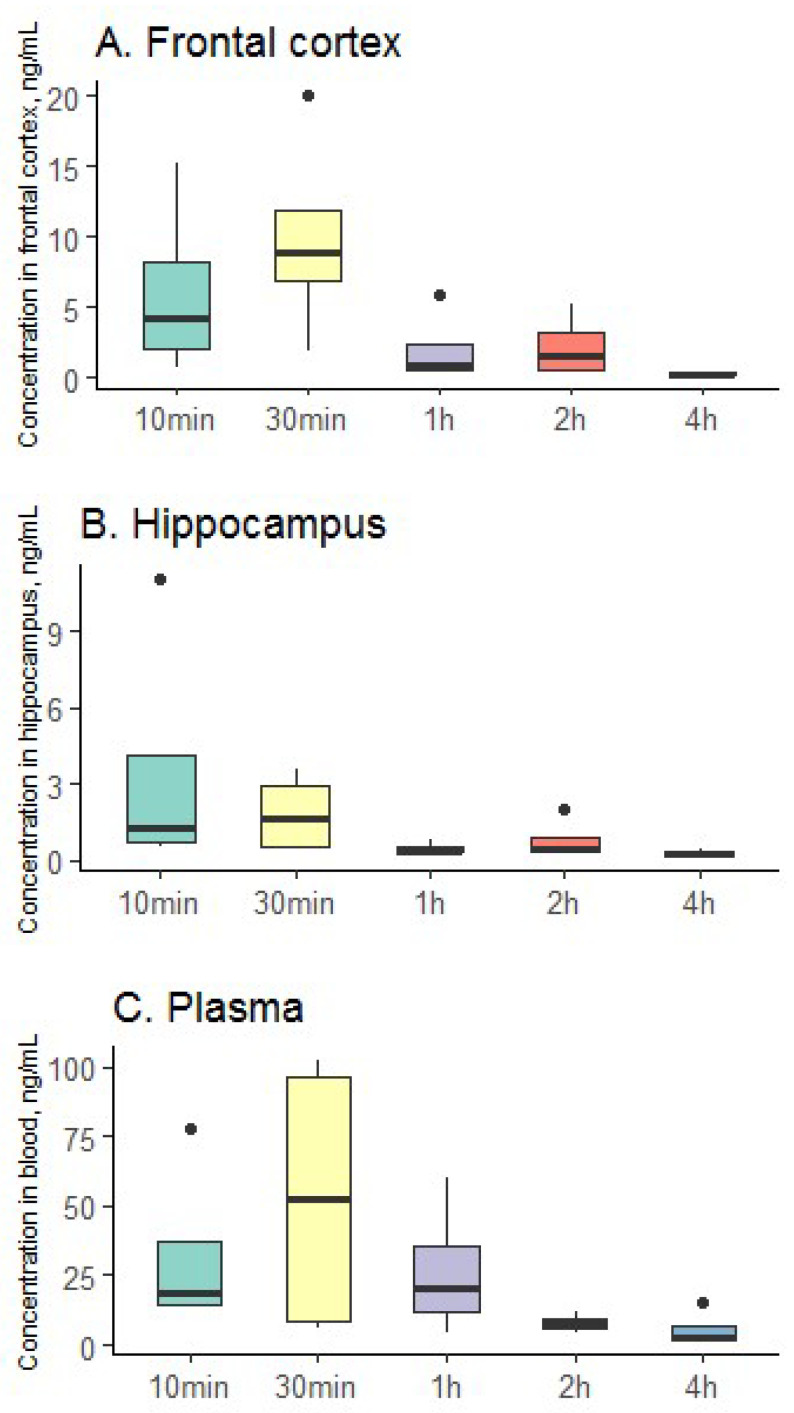
Distribution of clomipramine in brain tissue ((**A**)—frontal cortex; (**B**)—hippocampus and (**C**)—plasma); black circles—outliers.

The results of the study of pharmacokinetics in the blood and brain tissues of rats are shown in Figure 7.

According to the data obtained, it can be seen that the clomipramine-loaded protein aerogel can be successfully used for the delivery of the drug using the mechanism “Nose-to-brain”. Thus, in the hippocampus, the maximum concentration of clomipramine was reached already 10 min after the administration of the clomipramine-loaded protein aerogel. In the frontal cortex, the peak of drug concentration is observed after 30 min, as in the plasma of rats. The resulting clomipramine-loaded protein aerogel allows for the quick and efficient delivery of the drug to the brain and blood, which may contribute to the rapid onset of therapeutic effect.

## 3. Conclusions

In this paper, the results of a study of the process of obtaining protein aerogel particles based on egg white and whey protein isolate using various dispersion methods and analytical studies of the samples are presented. The research results have shown that the characteristic of protein aerogel particles can be set by changing the dispersion method during gelation, which makes it possible to obtain innovative aerogel-based materials with tailor-made properties. Experimental studies have been conducted to obtain a nasal drug delivery system. The clomipramine-loaded protein aerogel was obtained using an aerogel based on egg white and a drug—clomipramine—acting on the central nervous system. Analytical experiments were carried out to determine the amount and state of loaded clomipramine into the protein aerogel particles, the value of the mass loading of loaded clomipramine was determined by HPLC and amounted to 14.88 wt.%. The results of the X-ray analysis showed that in the resulting composition, clomipramine is predominantly in an amorphous state.

Experiments were conducted in vivo on the distribution of clomipramine in blood plasma and brain tissue of rats to study the pharmacokinetics and bioavailability of the resulting clomipramine-loaded protein aerogel. Protein aerogel partially reversed behavioral responses in rats that had been impaired by chronic stress. The peak concentration of clomipramine in the hippocampus was reached 10 min after the nasal administration of the composition. In the frontal cortex and plasma of rats, the peak concentration of drug was observed 30 min after administration of the clomipramine-loaded protein aerogel.

The results of this study demonstrate the effectiveness of protein aerogel particles as drug delivery systems. In particular, protein aerogel microparticles obtained by spraying are promising nasal drug delivery systems using the “Nose to brain” mechanism for the treatment of depressive disorders. Since even with a complex nasal form, protein aerogel particles in combination with a drug have achieved outstanding results as drug delivery systems, it can be concluded that by varying the size of protein aerogel particles, other drug delivery systems can be obtained, for example, for inhalation application.

## 4. Materials and Methods

### 4.1. Materials

To obtain protein aerogel particles the following materials were used: egg dry fermented protein (mass fraction of protein 85 wt.%, Roscar, Saint Petersburg, Russia) and whey protein isolate (mass fraction of protein 90 wt.%, Best Way Ingredients B.V., Haulerwijk, The Netherlands). Deionized water (Ravenol, Rostov-on-Don, Russia), isopropyl alcohol (>99.8 wt.%, Ruschim, Moscow, Russia), and carbon dioxide of high purity grade 4.0 were used as solvents. To obtain the drug-loaded protein aerogel, clomipramine hydrochloride powder (>98%, Sigma-Aldrich, Saint Louis, MO, USA) was used. To determine the amount of the adsorbed substance in the aerogel matrix, acetonitrile (>99.5%, Sigma-Aldrich, Saint Louis, MO, USA), tetramethylethylenediamine (Sigma-Aldrich, Saint Louis, MO, USA), and triethylamine (Sigma-Aldrich, Saint Louis, MO, USA) were used. The pH value was corrected with a solution of acetic acid (Ruschim, Moscow, Russia).

### 4.2. Synthesis of Protein Aerogel Particles

**Figure 8:**
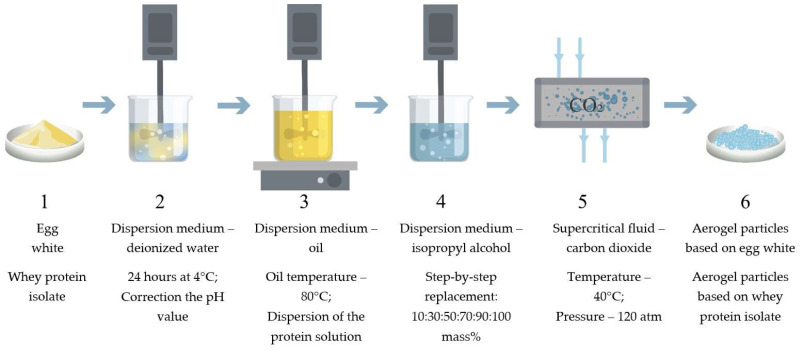
Scheme of the synthesis of protein aerogel particles: (**1**) initial protein; (**2**) obtaining protein solution; (**3**) dispersion and gelation; (**4**) oil washing and solvent replacement; (**5**) supercritical drying; (**6**) protein aerogel particles.

The process of obtaining protein aerogel particles includes the following main stages: obtaining a protein solution and correcting the pH value (Figure 8 stage 2), dispersing the protein solution into oil and thermal gelation (Figure 8 stage 3), washing the formed gel particles from the oil and step-by-step solvent exchange with isopropyl alcohol (Figure 8 stage 4), and supercritical drying (Figure 8 stage 5). Figure 8 shows the main stages of obtaining protein aerogel particles.

**Figure 9:**
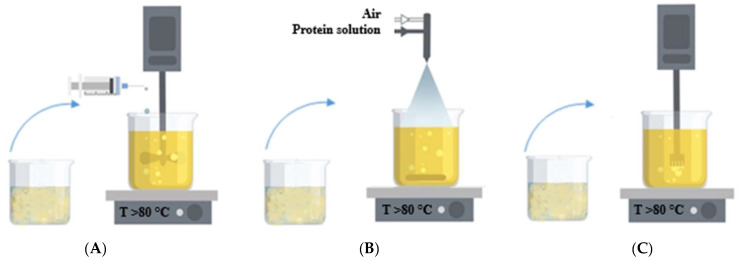
Dispersing methods of the protein solution into oil: (**A**)—dripping; (**B**)—spraying through a nozzle; (**C**)—homogenization.

In this work, the type of protein (egg white and whey protein isolate) was varied, as well as the dispersion method of the protein solution into oil, i.e., the method of forming a protein gel (drip, spraying, and homogenization). Figure 9 shows the methods of dispersing the protein solution into oil.

The variable parameters of the process of obtaining protein aerogel particles are presented in Table 2.

#### 4.2.1. Obtaining a Protein Solution

A given amount of dry fermented protein was dissolved in deionized water and stirred at room temperature. The protein solution was then stored for 12 h at a temperature of 5 °C to ensure complete dissolution of the protein. The pH value of the protein solution was adjusted to the set value for all samples

#### 4.2.2. Obtaining Protein Gel Particles by Dripping Method

Sunflower oil was heated in a water bath to 80–85 °C. Mixing was carried out by a four-bladed top-drive agitator (IKA EUROSTAR) at a rotation speed of 500 rpm. The protein solution was added to sunflower oil in a volume ratio of 1:3. The protein solution was dispersed drop by drop into sunflower oil using a digging device. In the process of continuous mixing, protein gel particles were formed in the oil. After, the gel particles were kept in sunflower oil at a temperature of 80–85 °C for at least 10 min to complete the gelation process and prevent agglomeration of the particles.

#### 4.2.3. Obtaining Protein Gel Particles by Spraying

Sunflower oil was heated in a water bath to 80–85 °C. Mixing was carried out by a magnetic stirrer at a rotation speed of 300 rpm. The protein solution was added to sunflower oil in a volume ratio of 1:3. For the spraying process, a pneumatic external mixing nozzle manufactured by Glatt GmbH with an internal diameter of 0.5 mm was selected. Parameters of the spraying process: the consumption of the protein solution is 21.0 mL/min, and the distance from the nozzle to the surface of sunflower oil was 10 sm. Upon contact of drops of protein solution with sunflower oil, the process of gel particles formation took place. After, the protein gel particles were kept in sunflower oil at a temperature of 80–85 °C for at least 10 min to complete the gelation process and prevent agglomeration of the particles.

#### 4.2.4. Obtaining Protein Gel Particles by Homogenization

Sunflower oil was heated in a water bath to 80–85 °C. Mixing was carried out using a dispersant (IKA T25 digital ULTRA-TURRAX) at a rotor speed of 4000 rpm. The protein solution was added to sunflower oil in a volume ratio of 1:3. In the process of continuous mixing, particles were formed. When the drops of the protein solution got into the oil, the gelation process began. After, the gel particles were kept in sunflower oil at a temperature of 80–85 °C for at least 10 min to complete the gelation process and prevent agglomeration of the particles.

#### 4.2.5. Solvent Exchange

To prepare the gel for supercritical drying, it is necessary to replace the solvent inside the gel. Isopropyl alcohol was chosen for this purpose. In order to avoid deformation of the structure during the solvent replacement, the concentration of isopropanol was gradually increased. The concentration of isopropanol was 10%, 30%, 50%, 70%, 90%, 100%, and 100%. To completely replace the solvent inside the pores of the gel, it is necessary to place the gel in an isopropanol solution with a given concentration for at least 2 h. As a result, protein gel particles prepared for the supercritical drying stage were obtained.

#### 4.2.6. Supercritical Drying of Protein Gel Particles

**Figure 10:**
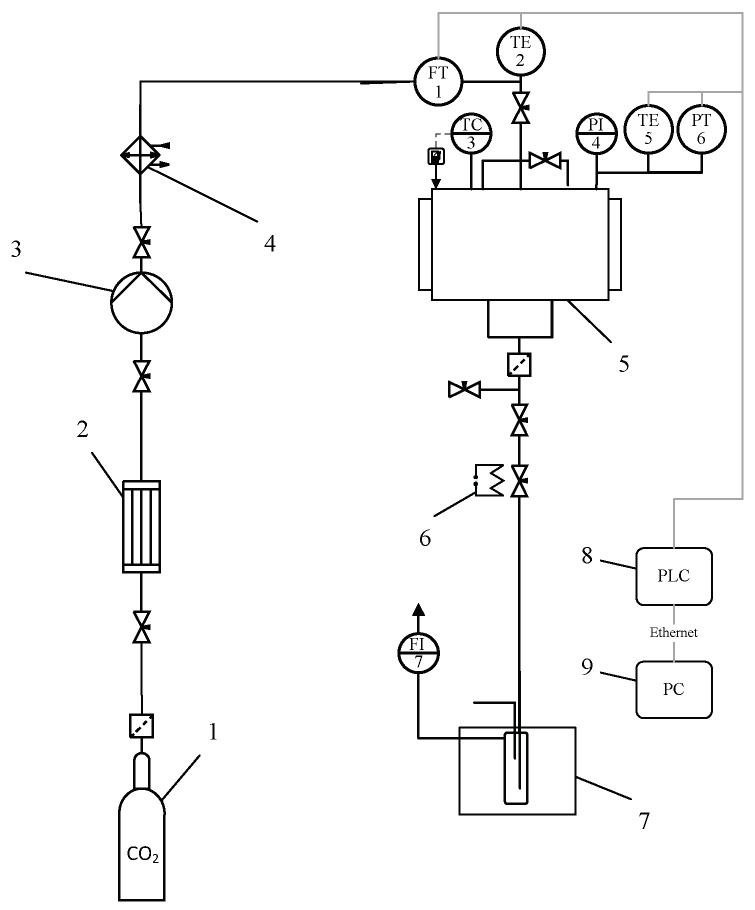
Supercritical drying experimental setup: 1—vessel with liquid CO_2_ (60 bar); 2—condenser; 3—pneumatic high-pressure pump; 4—thermostat; 5—250-mL high-pressure apparatus; 6—decompression valve with heating; 7—separator with cooling; 8—programmable logic controller (PLC); 9—personal computer (PC); TC3—temperature controller; FT1—Coriolis flow meter; TE2, TE4—thermocouples; PT5—pressure sensor.

Protein aerogel particles were obtained using supercritical drying. During the drying of protein gels, diffusion substitution of isopropyl alcohol from the porous structure of the protein gel to supercritical carbon dioxide is carried out without the formation of a phase interface, followed by a decrease in pressure and the transition of carbon dioxide to a gaseous state, as a result of which the original structure of the protein gel is preserved. The supercritical drying experimental setup of protein gel is shown in Figure 10.

Parameters of the supercritical drying process: carbon dioxide consumption—0.2 kg/h, supercritical fluid—carbon dioxide, temperature—40 °C, and pressure—120 atm for 6 h.

#### 4.2.7. Preparation of the Clomipramine-Loaded Protein Aerogel

In the course of the work, sample 3 was used for the development of a nasal drug delivery system, and the substance clomipramine was used for the loading into the aerogel. The choice of sample 3 is due to the presence of a high specific surface area of and relevant aerodynamic particle diameter [7]. Clomipramine is an antidepressant that has an antihistamine and alpha–adrenoblocking effect, the drug is used to prevent and combat the manifestation of depressive states. Clomipramine is very easily soluble in water and alcohol [41]. There is no information on the solubility of clomipramine in supercritical carbon dioxide. Based on the physicochemical properties of clomipramine, the method of adsorption from a solution was chosen for its load into the protein aerogel. This method allows for the achievement of the necessary mass loading of the drug into the aerogel, as well as maintenance of the stability of the drug during implementation due to the mild conditions of the process.

The process of the loading of clomipramine into the protein aerogel was carried out using the method of adsorption from a solution. The adsorption process consisted of the following steps: a sample of protein aerogel of a given mass (1.17 g) was in contact with a previously obtained solution of clomipramine of a given concentration (146 ×·10^−4^ g/mL). In the process, clomipramine diffused from the solution into the pores of protein aerogel particles.

### 4.3. Analytical Studies

The morphology of the surface of the obtained aerogel particles was studied by scanning electron microscopy (SEM) on a JEOL 1610LV device. The determination of the skeletal density, specific surface area, average pore size, and SEM images was carried out at the Center for Collective Use of the D.I. Mendeleev Russian University of Chemical Technology.

The structural characteristics of protein aerogel particles, namely, the specific surface area, volume, and pore diameter, were studied by low-temperature N_2_ adsorption–desorption analysis (at −196 °C) on the specific surface and porosity analyzer ASAP 2020 MP. The specific surface area was determined by the Brunauer–Emmett–Teller (BET) method; the volume of pores and their average diameter was determined by the Barrett–Joyner–Halenda (BJH) method. The determination of the skeletal density was carried out by the pycnometric method on the helium pycnometer “AccuPyc II 1340” (Micromeritics). The bulk density of the protein aerogel was calculated by the Formula (1):

(1) $$\rho_{bulk}=\frac{m_{particles}}{V_{particles}}$$

where, *ρ_bulk_*—bulk density, g/sm^3^; *m_particles_*—particle mass, g; *V_particles_*—particle volume, sm^3^.

Porosity of protein aerogel particles was calculated by the Formula (2):

(2) $$P=(1-\frac{\rho_{\mathrm{skeletal}}}{\rho_{\mathrm{bulk}}})\times 100,$$

where, *P*—aerogel porosity, %; *ρ*_bulk_—bulk density, g/sm^3^, *ρ_s_*_keletal_—skeletal density, g/sm^3^.

Experiments to obtain the size distribution of protein gel particles were carried out by laser diffraction. The measurements were carried out on the Analysette 22 NanoTec plus laser particle size analyzer. For samples obtained by the dripping method (1 and 4), particle size measurements and data processing were carried out using an optical microscope Micros Austria and Imaje J program. According to the obtained differential curves of particle size distribution, the average diameter of protein particles was determined. The average diameter of the particles was calculated by the Formula (3):

(3) $$d=\underset{i}{\sum }\frac{n_{i}}{\sum_{i}n_{i}}=\underset{i}{\sum }f_{ni}d_{i}$$

where, *n_i_*—the number of particles in the *i*-th fraction (diameter *d_i_*); ∑*_I_ n_i_*—the total number of particles in the system; *f_ni_* = *n_i_*/∑*_j_ n_j_*—numerical fraction of the *i*-th fraction.

Since aerogel particles have a low density and high porosity, their aerodynamic diameter should be taken into account to select the most effective drug delivery system [42,43,44]. Therefore, the aerodynamic diameter was calculated for all samples according to Formula (4):

(4) $$d_{a}=d*\sqrt{\rho_{bulk}*\left(1-\varepsilon \right)}$$

where, *ρ_bulk_*–bulk density, g/sm^3^; *ε*—layer porosity, sm^3^/sm^3^. The standard porosity of the powder layer is 0.4.

All experimental points were removed at least three times. The average results of analytical studies are shown in Table 1.

The amount of loaded clomipramine into the pores of the protein aerogel was determined by high-performance liquid chromatography on the Agilent 1220 device. The quantity of the adsorbed substance was determined by high-performance liquid chromatography. The mobile phase for clomipramine was obtained by the following method: acetonitrile: buffer in a volume ratio of 37:63. Buffer composition: 0.5 vol% tetramethylethylenediamine and 0.01 vol% triethylamine; pH 6.5 was regulated with a solution of acetic acid. Then, the resulting solution was left in an ultrasonic bath for half an hour. Detection conditions: volumetric flow rate of the mobile phase—1.5 mL/min; wavelength—254 nm; sample volume—20 µL [45].

The presence of crystalline or amorphous phases of clomipramine in the clomipramine-loaded protein aerogel was determined by qualitative X-ray analysis on an InelEquinox 2000 powder X-ray diffractometer.

### 4.4. In Vivo Evaluations

#### 4.4.1. Animals

The male Wistar rats (2 months old and 200–250 g weight) were obtained from the Nursery for Laboratory Animals (Pushchino, RAS, Moscow, Russia). Animal housing condition were as follows: constant temperature of 23 °C, controlled direct lighting (12/12 h), water and food access ad libitum. During stress protocols the rats were housed in individual transparent cages of polycarbonate (42 × 26 × 15 cm). After the stress exposure, the animals were placed in cages of 8 rats each. All procedures with the animals were maintained in accordance with Directive 2010/63/EU of 22 September 2010. The animal study protocol was approved by the local ethical committee of V.P. Serbsky National Medical Research Centre of Psychiatry and Narcology (protocol No. 1 from 17 February 2022).

#### 4.4.2. Experiment Design with Behavior

The animals were divided into the following experimental groups: control animals (*n* = 8); rats exposed to chronic stress for 3 weeks (*n* = 8); and rats exposed to chronic stress for 3 weeks with protein aerogel nasal treatment (*n* = 8). On the next day after the end of the chronic stress protocol, the sucrose preference test was performed. The behavior tests were conducted with a delay of 1 day between them in the following order: sucrose preference test, object recognition test, and forced swim test. The interval between tests was at least two days.

#### 4.4.3. Distribution of Drugs in Brain Tissue

A special experiment was conducted to determine the distribution of clomipramine in the brain. Brain samples were collected 10 min, 30 min, 1, 2, and 4 h after drug administration. There were 4 different rats for each point. Brain sections were taken from the same rats. Brain sections were taken on cold glass, frozen in liquid nitrogen, and stored at −20 °C.

#### 4.4.4. Chronic Stress

A depressive-like state of rodents was induced by exposure to ultrasonic waves of variable frequencies. Ultrasonic frequencies of 20–45 kHz were applied continuously for 21 days using a Weitech ultrasonic generator (Wavre, Belgium). The sound pressure supplied by the ultrasonic generator was 50 dB ± 5 dB (fluctuation ± 10%). The frequency ranges of ultrasound stimulation alternated every 10 min between the following intervals: short frequencies (20–25 kHz), medium frequencies (25–40 kHz), and high frequencies (40–45 kHz). In general, the total duration of the radiation at short and medium frequencies was 35%, at high frequencies—30%. During the application of radiation, the animals were in individual cages, the location of the cages changed daily. The drugs were administered every day for 21 days of stress [46,47,48].

#### 4.4.5. Drug Administration

During the exposure to the USA, we administered intranasal protein aerogel with a drug load (dosage 7.5 mg/kg) to rats daily at 10 a.m. Each rat was held belly up and aerogel was administrated intranasal into each nostril. Other groups received intranasal saline in the same volume for the same number of days. Clomipramine was administered in the same dose once to study drug distribution in the blood and brain [49].

#### 4.4.6. Sucrose Preference Test

We gave the rats two bottles, one with a 1% sucrose solution and one with tap water. The test lasted 24 h. We started the test in the evening (active phase of rats). We weighed bottles before and after the test. Then, we calculated consumption. To prevent place preference, we changed the position of the bottles. The formula for sucrose index is: Sucrose Preference = Volume (Sucrose Solution)/(Volume (Sucrose solution) + Volume (Water) × 100 [46].

#### 4.4.7. Forced Swim Test

The test was performed in a round pool (diameter—31 cm, height—40 cm). The water temperature was 23 °C. We put the rat into the pool for 8 min. In the first 2 min we did not record video. We considered immobility as the absence of movements of the head and limbs. Immobility was evaluated using a digital camera and RealTimer software Version 1.30 (OpenScience, Moscow, Russia) [47].

#### 4.4.8. Object Recognition Test

We prepared the novel object recognition test in a gray plastic arena (45 cm × 45 cm × 40 cm). Tests were performed for 3 consecutive days. Each experiment lasted for 5 min with a 24 h interval. The first day: we tested animals without any objects. Second day: we tested animals with two identical objects. The third day: one object was replaced with a non-identical object. The formula for discrimination index is: (TN − TF/TN + TF) × 100%. TN—novel object time recognition, TF—old object time recognition. We excluded the animals which explored objects for less than 10 s [50].

### 4.5. Sample Preparation for the Determination of Clomipramine in Blood Plasma and Brain Tissues

#### 4.5.1. Sample Preparation of Rat Blood Plasma

Prior to the analysis, samples were stored at −80 °C. Samples were thawed on ice. After defrosting, the samples were placed on the vortex for 10 s. An aliquot of 200 µL of plasma was taken from each rat blood plasma sample in a separate labeled Eppendorf. A total of 400 µL of cooled methanol (−80 °C) was added to the sample, which contained an internal standard. The samples were placed on a shaker for 10 min at 1500 rpm at 10 °C. The obtained samples were centrifuged at maximum speed for 10 min, after which 400 mL of the supernatant was selected. The resulting sample was lyophilized dry, after which the precipitate was reconstructed into 50 mL of a water–methanol mixture (80:20) and placed on the vortex for 20 s. The reconstructed sample was subjected to centrifugation at maximum speed for 10 min at 10 °C. A total of 35 mL of the supernatant was taken from the samples, transferred to pre-marked vials with inserts, hermetically sealed, and transferred for analysis.

#### 4.5.2. Sample Preparation of Rat Brain Tissues

Samples were stored at −80 °C prior to analysis. A pre-weighted sample of rat brain tissue was placed in a pre-filled homogenization vial. A total of 500 mL of a cooled water–methanol mixture (20:80) containing an internal standard was added to the suspension. The samples were placed on a shaker for 10 min at 1500 rpm at 10 °C. The hitch was homogenized for 20 s using a homogenizer. The resulting sample was centrifuged at 3000 rpm for 2 min, after which 400 mL of the supernatant was taken. The supernatant was repeatedly centrifuged at maximum rpm for 10 min at 10 °C. Then, the sample was lyophilized to dry, after which the precipitate was reconstructed into 50 mL of a water–methanol mixture (20:80) and placed on the vortex for 20 s. The reconstructed sample was subjected to centrifugation at maximum speed for 10 min at 10 °C. A total of 35 mL of the supernatant was taken from the samples, transferred to pre-marked vials with inserts, hermetically sealed, and transferred for analysis.

A tandem SCIEX 4500QTRAP mass spectrometer combined with a Shimadzu 30ACMP liquid chromatograph was used for the analysis. Source settings: GS1 = 55; GS2 = 55; CUR = 35; TEM = 450 C; ISFV = 5500 V; an ESI probe was used to feed the sample. Amitriptyline has been used as an internal standard. The detection of ions of the studied substances was carried out in the monitoring mode of multiple reactions (Table 3).

Chromatographic separation of the components of the test sample was carried out in RPLC mode using a chromatographic column Phenomenex Phenyl-Hexyl 2.1 × 50 mm 2.6 um: phase A (water; 5 mM ammonium formate); phase B (ACN; 5 mM ammonium formate); flow rate 0.45 mL/min; sample input volume 1 µL. To switch the chromatograph flow between the mass spectrometer and the drain, an integrated tap of the Sciex 4500QTRAP mass spectrometer was used; in the range of 2.3–3.1 min, the solvent flow was fed into the mass spectrometer source. The chromatographic gradient conditions are shown in Table 4. The calibration curve for clomipramine was constructed in the range of 0.06–250 ng/mL.

The quality control system included the insertion of quality control samples (QC) before and after the end of the analytical series, as well as every 10 samples of the analytical series. The result of the analytical series was considered satisfactory if the difference between the chromatographic peak area for quality control samples during the analytical series did not exceed 10%. In addition, quality control samples were used to estimate the value of the cross-transfer of the sample by evaluating the response for the internal standard after stabbing the sequential insertion of a quality control sample and a blank sample every 10 samples of the analytical series. A cross-transfer value of less than 0.1% was considered acceptable. Skyline and Sciex Analyst software were used to quantify the compounds under study. An example of the obtained chromatograms is shown in Figures S1–S4 and are presented in the Supplementary Materials.

## Figures and Tables

**Table 1:** Characteristics of the protein aerogel.

Sample	S_BET_ (m^2^/g)	V_BJH_ (sm^3^/g)	D_pore_ (nm)	ρ_skeletal_ (g/sm^3^)	ρ_bulk_ (g/sm^3^)	P (%)	d (µm)	d_a_ (µm)
1	275	1.6	23	1.333	0.206	85	830	292
2	346	2.9	34	1.428	0.221	85	67	24
3	310	2.1	28	1.797	0.116	94	228	60
4	72	0.4	20	1.237	0.097	92	892	215
5	152	0.6	17	1.660	0.239	85	184	70
6	56	0.4	25	1.464	0.074	96	166	35

**Table 2:** Parameters of the process of obtaining protein aerogel particles.

No.	Type of Protein	Concentration (wt.%)	pH	Dispersion Method
1	Egg	15	7	Dripping
2				Spraying
3				Homogenization
4	Whey isolate	20	9	Dripping
5				Spraying
6				Homogenization

**Table 3:** Multiple-reaction monitoring (MRM) transitions for the studied substances.

Substance	Q1	Q3	Dwell Time	DP	CE	EP	CXP
Clomipramine	315.16	86.10	40	26	23	10	12
Clomipramine	315.16	58.06	40	26	67	10	16
Amitriptyline	278.19	233.15	25	70	23	10	12
Amitriptyline	278.19	91	25	70	27	10	7

**Table 4:** Conditions of chromatographic analysis.

Time [min]	Flow [mL/min]	B. Conc [%]	B. Curve
0.15	0.4	15	0
2.5	0.4	55	0
2.7	0.4	99	0
3.5	0.4	99	0
3.55	0.4	15	0
4.55	0.4	15	0

## Supplementary Materials

The following supporting information can be downloaded at: https://www.mdpi.com/article/10.3390/gels8120765/s1, Figure S1: Chromatogram for the point of the calibration curve equal to the lower limit of quantitative determination (LOQ) for clomipramine (6 pg/mL); Figure S2: Chromatogram for the point of the calibration curve equal to the upper limit of quantitative determination (LOQ) for clomipramine (250 ng/mL); Figure S3: Chromatogram for clomipramine and internal standard for sample 20220530_RB_Cla_S1_RBC_077_rep 001; Figure S4: Chromatogram for clomipramine and internal standard for sample 20220530_RB_Cla_S1_BC_017_rep 001.
