# Supplementary material for: Preparation of Protein Aerogel Particles for the Development of Innovative Drug Delivery Systems
Source: Gels. 2022 Nov 24;8(12):765. doi: 10.3390/gels8120765 (PMC9777701; doi:10.3390/gels8120765)
Supplement: Supplementary file 1 [file gels-08-00765-s001.zip › gels-2052969-supplementary.pdf]

Supplementary materials

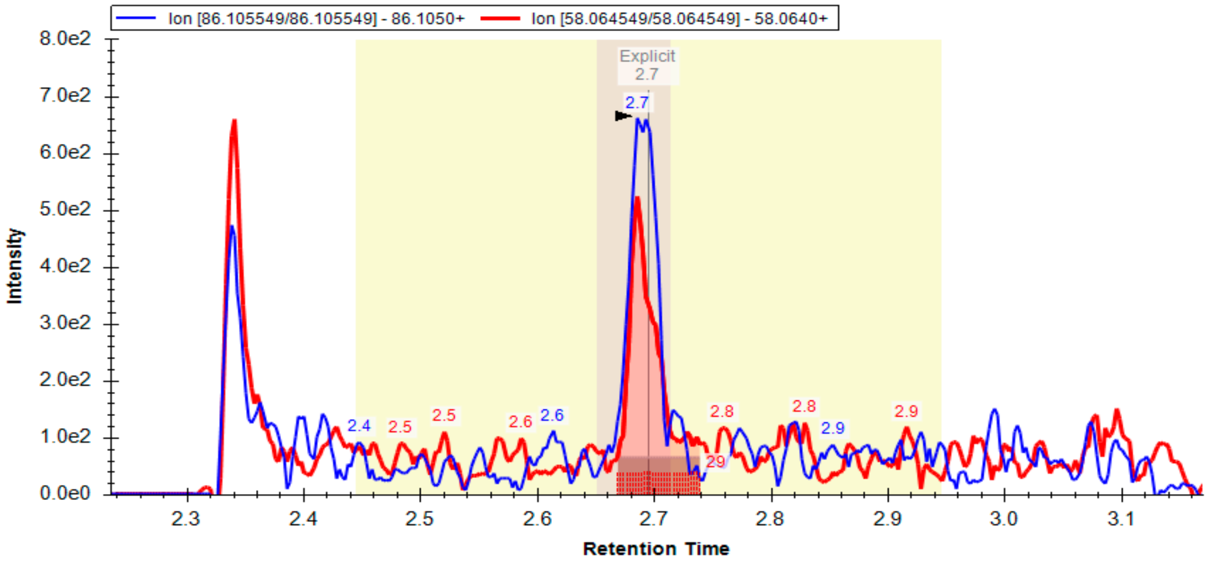

Figure S1. Chromatogram for the point of the calibration curve equal to the lower limit of quantitative determination (LOQ) for clomipramine (6 pg/ml).

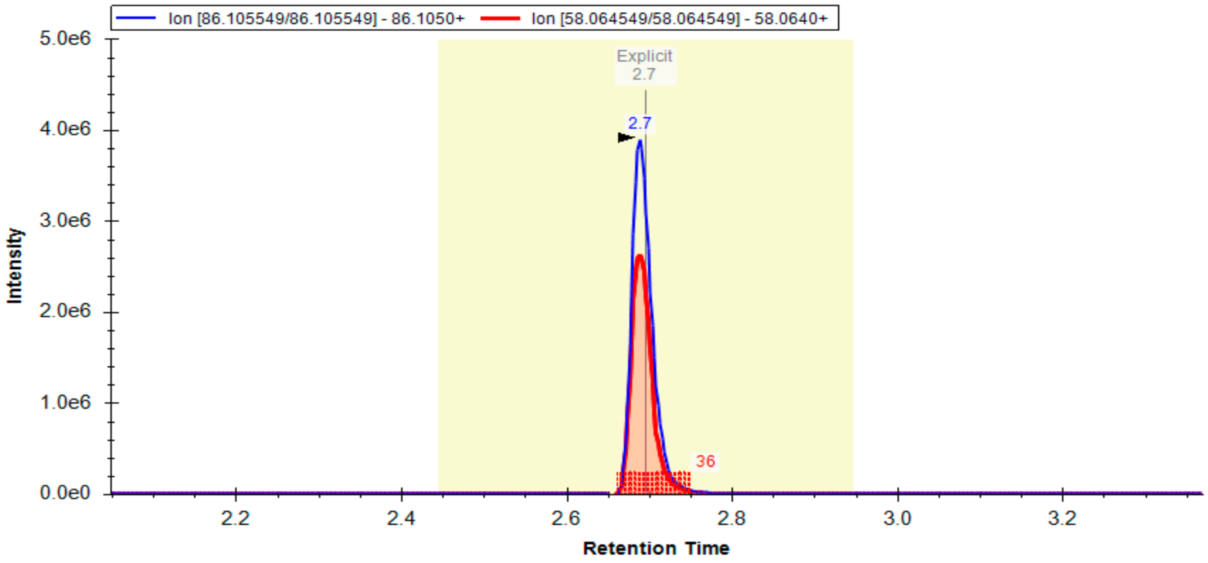

Figure S2. Chromatogram for the point of the calibration curve equal to the upper limit of quantitative determination (LOQ) for clomipramine (250 ng/ml).

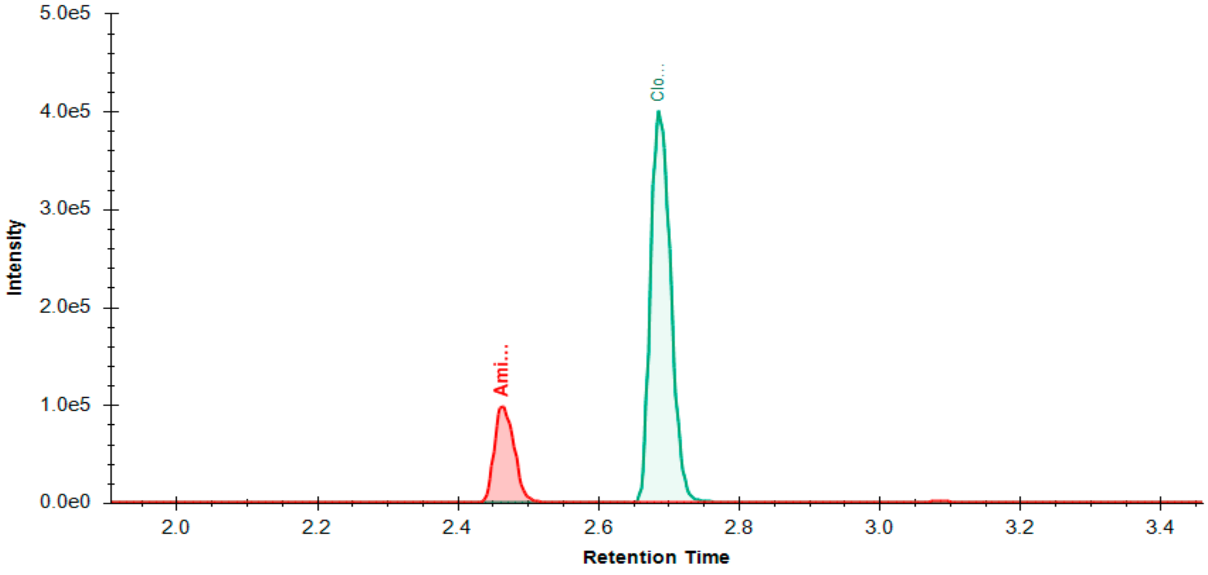

Figure S3. Chromatogram for clomipramine and internal standard for sample 20220530_RB_Cla_S1_RBC_077_rep 001.

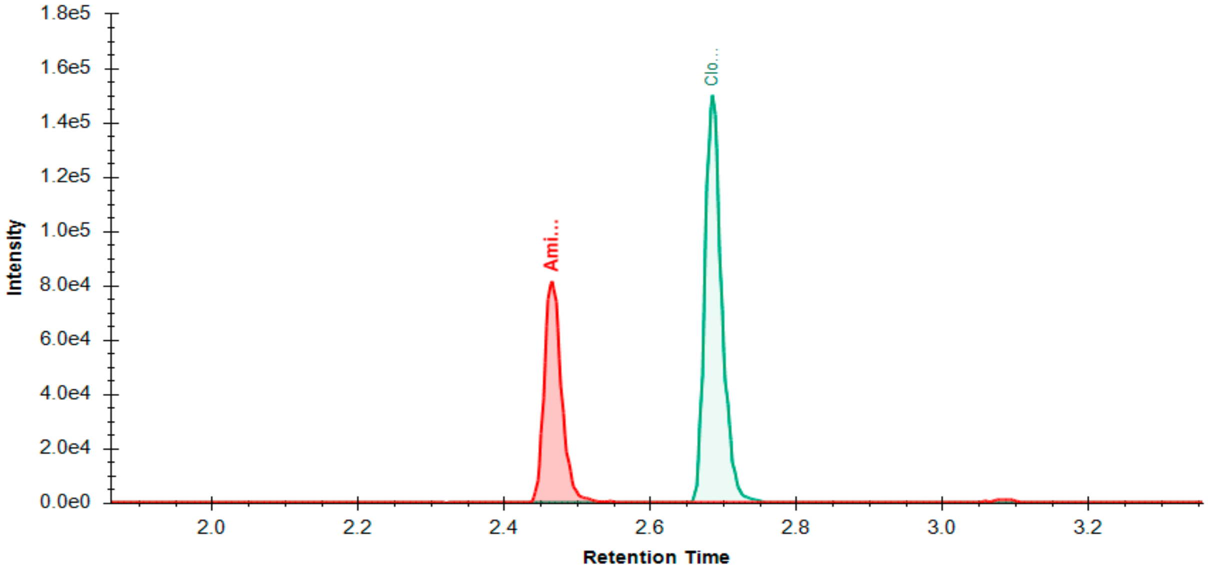

Figure S4. Chromatogram for clomipramine and internal standard for sample 20220530_RB_Cla_S1_BC_017_rep 001.
